# Overwhelming Asplenic Sepsis due to Babesiosis

**DOI:** 10.4274/tjh.galenos.2019.2019.0080

**Published:** 2019-11-18

**Authors:** Chakra P. Chaulagain

**Affiliations:** 1 Department of Hematology-Oncology, Myeloma and Amyloidosis Program, Maroone Cancer Center, Cleveland Clinic Florida, Weston, FL, USA

**Keywords:** Babesiosis, Splenectomy, Sepsis

## To the Editor,

**Figure 1:**
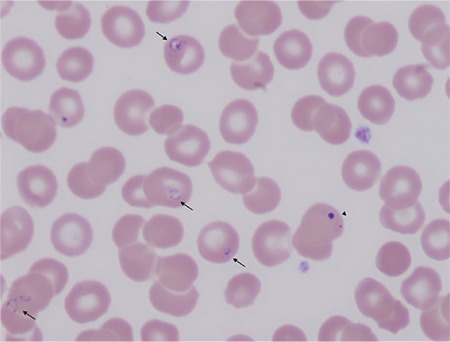
Thin blood smear with oil immersion showed intraerythrocytic polymorphic ring forms (arrows) morphologically consistent with *Babesia* species and the presence of Howell-Jolly bodies (arrowhead), confirming the history of splenectomy.

A 70-year-old female from southern Massachusetts, USA, was admitted to the intensive care unit with septic shock and acute respiratory distress syndrome (ARDS) after 3 days of acute febrile illness. She had undergone splenectomy at the age of 5 related to trauma from a traffic accident. Laboratory studies reveled pancytopenia, acute renal insufficiency, increased lactate dehydrogenase, depressed haptoglobin, and elevated liver enzymes with indirect hyperbilirubinemia. Prothrombin time and activated partial thromboplastin time were both elevated and fibrinogen level was low, consistent with disseminated intravascular coagulation (DIC). A direct anti-globulin test was negative. A thin blood smear with oil immersion showed intraerythrocytic polymorphic ring forms (Figure 1, arrows) morphologically consistent with *Babesia* species and the presence of Howell-Jolly bodies (Figure 1, arrowhead), confirming the history of splenectomy. Real-time DNA-PCR confirmed *Babesia microti* as the offending parasite. The patient was started on treatment for babesiosis with quinine, azithromycin, and atovaquone. She also received red cell exchange transfusion due to the high level of parasitemia (14% of the erythrocytes) and completely recovered in the next few weeks.

Human babesiosis is a malaria-like tick-borne illness caused by the protozoan parasite *Babesia microti*, endemic in the Midwest and Northeast USA; it has also been reported in parts of Europe, Asia, and Australia [1]. It has also been reported after transfusion of contaminated blood products [2]. Infection is usually mild to moderate in an immunocompetent host but a severe infection requiring hospitalization can occur in patients with a history of splenectomy or immunodeficiency such as cancer, human immunodeficiency virus infection, or hemoglobinopathy and in the elderly with co-morbidities and allogeneic hematopoietic stem cell transplant recipients [1,3]. Severe babesiosis with ARDS and DIC can occur in immunocompromised or asplenic individuals, which can be fatal [4,5]. Milder illness in immunocompetent hosts manifests with malaise, fever, headache, myalgia, and nausea. Laboratory findings typically show non-immune hemolytic anemia and thrombocytopenia, but immune hemolytic anemia has also been reported. A rapid diagnosis can be made by identification of *Babesia *organisms on thin blood smears under oil immersion. The diagnosis can be confirmed by using DNA-PCR to identify the DNA of the parasite. Serology is available, but it is difficult to distinguish current from recent or past infection in a patient coming from an endemic area. The most commonly used agents for treatment of severe babesiosis include azithromycin, atovaquone, quinine, and clindamycin. Patients with severe infection with high-grade parasitemia, severe hemolysis, or compromised organ functions (pulmonary, liver, or renal impairment) may benefit from red cell exchange transfusion.

This case confirms that a severe form of babesiosis can occur in patients who have undergone splenectomy. A high index of suspicion and a timely review of blood smears in asplenic patients presenting with febrile illness and hemolytic anemia from endemic areas can aid in rapid diagnosis and prompt treatment, which can be lifesaving. This case illustrates that an early diagnosis and aggressive treatment can be lifesaving even with a fulminant and severe infection with babesiosis. The key is to quickly decrease the parasitic burden for a good clinical outcome.
